# Azulene and Its Derivatives as Potential Compounds in the Therapy of Dermatological and Anticancer Diseases: New Perspectives against the Backdrop of Current Research

**DOI:** 10.3390/molecules29092020

**Published:** 2024-04-27

**Authors:** Emilia Slon, Bartosz Slon, Dorota Kowalczuk

**Affiliations:** 1 Chair and Department of Medicinal Chemistry, Faculty of Pharmacy, The Medical University of Lublin, Jaczewskiego 4, 20-090 Lublin, Poland; dorota.kowalczuk@umlub.pl; 2 A-Sense Sp. z o.o., Moscickiego 1, 24-100 Pulawy, Poland; bartosz.slon@a-sense.pl

**Keywords:** azulene, guaiazulene, chamazulene, derivatives, dermatological diseases

## Abstract

The scientific article focuses on the role of azulene and its derivatives in the therapy of dermatological diseases, presenting the latest laboratory and clinical research as well as prospects for further studies. In a synthetic literature review, various databases such as PubMed, Scopus, Web of Science, and the Database of Polish Scientific Journals were queried to select relevant articles concerning azulene. The conclusions drawn from the thematic analysis of the studies emphasize the multifaceted pharmacological actions of azulene and its derivatives including their anti-inflammatory properties, potential anticancer effects, photoprotective abilities, alleviation of itching, management of atopic dermatitis, and treatment of erectile dysfunction. However, there are certain limitations associated with the application of unmodified azulene on the skin, particularly related to photodecomposition and the generation of reactive oxygen species under UV radiation. These effects, in turn, necessitate further research on the safety of azulene and azulene-derived substances, especially regarding their long-term use and potential application in phototherapy. The authors of this work emphasize the necessity of conducting further preclinical and clinical studies to fully understand the mechanisms of action. Incorporating azulene and its derivatives into the therapy of dermatological disorders may represent an innovative approach, thereby opening new treatment avenues for patients.

## 1. Introduction

**Figure 1:**
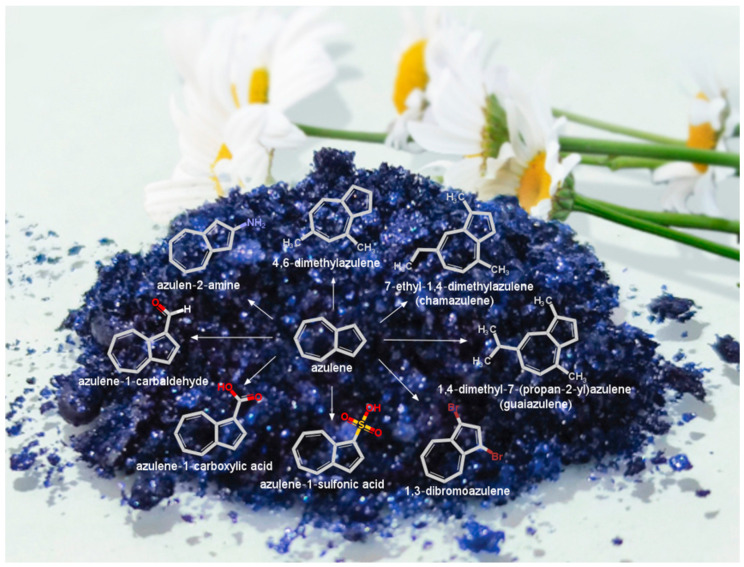
Azulene and its basic derivatives.

Contemporary dermatology constantly seeks innovative solutions that can effectively improve the quality of life for patients suffering from various skin disorders. One promising avenue of research is the application of azulene and its derivatives in the therapy of dermatological diseases. Azulene, characterized by its distinctive blue color, is composed of a seven-membered cycloheptatriene ring fused with a five-membered cyclopentadiene ring, unlike its colorless isomer—naphthalene. Azulene is mainly found in natural essential oils of plant origin including *Matricaria chamomilla* L. (*M. chamomilla*), *Achillea millefolium* L. (*A. millefolium*), and *Guaiacum officinale* L. (*G. officinale*). *G. officinale* also contains chamazulene and guaiazulene. Other derivatives of azulene include methyl (7-isopropenyl-4-methylazulen-1-yl) stearate found in the fungus *Lactarius indigo* (*L. indigo*) and 7-acetylo-1,4-dimethylazulene present in the fungus *Entoloma hochstetteri* (*E. hochstetteri*) as well as 2,3-dihydrolinderazulene and linderazulene in marine invertebrates—gorgonians *Acalycigorgia* sp. [1,2,3,4,5,6]. In recent years, we have observed increased interest among researchers in azulene and its derivatives, particularly in the context of dermatological disease therapy. Basic modifications involve the substitution of hydrogen in the azulene structure with an appropriate substituent. The most common substituents include alkyl groups such as methyl (-CH_3_), ethyl (-C_2_H_5_), or isopropyl (-C_3_H_7_) as well as alkoxyl groups such as methoxyl (-OCH_3_), halogens, and functional groups such as amino (-NH_2_), carboxyl (-COOH), aldehyde (-CHO), hydroxyl (-OH), or others (Figure 1) [7,8,9,10,11,12,13].

Laboratory and clinical studies confirm the ability of azulene to inhibit inflammatory processes at the cellular level, which opens up new therapeutic perspectives.

In this paper, we will present a review of the latest research concerning azulene and its derivatives as potential compounds in the therapy of dermatological diseases and anticancer, analyzing both the mechanisms of action and the results of clinical studies. Additionally, we will discuss potential challenges and new development opportunities for this promising family of active substances, highlighting possible directions for further research. We believe that tracking progress in azulene research is worthwhile, as it may contribute to advancements in the treatment of skin diseases and potentially cancer, opening up new therapeutic possibilities for patients.

## 2. Materials and Methods

To identify studies focusing on the application of azulene derivatives, a comprehensive approach was adopted. A systematic literature search was executed across four electronic databases, namely PubMed, Scopus, Web of Science, and the Database of Polish Scientific Journals. Specific search terms encompassing variations of “azulene”, or “guaiazulene”, or “chamazulene”, or “azulene derivatives” were combined with terms related to “anti-cancer activity”, “cancer”, or “dermatological diseases”, and “skin”. Studies published between 2003 and 2024 were considered within the search criteria, which was conducted exclusively in English.

The initial search across the aforementioned databases yielded a total of 248 studies. Subsequently, the removal of duplicates from selected databases and the application of filters based on article type, title, and abstract resulted in the identification of forty five studies that were directly pertinent to the objective of this work.

## 3. Mechanisms of Anti-Inflammatory Action

Azulene demonstrates the ability to exert anti-inflammatory effects through complex interactions with biological pathways. One of the key mechanisms involves the inhibition of the enzyme cyclooxygenase-2 (COX-2), which participates in the biosynthesis of prostaglandins, key mediators of inflammatory processes. It also exhibits the ability to regulate the secretion of cytokines, proteins that play a significant role in transmitting immunological signals. Another aspect of azulene’s mechanism of action is the stabilization of cell membrane structures, leading to reduced susceptibility to damage and limiting the body’s inflammatory response [5,14,15].

**Figure 2:**
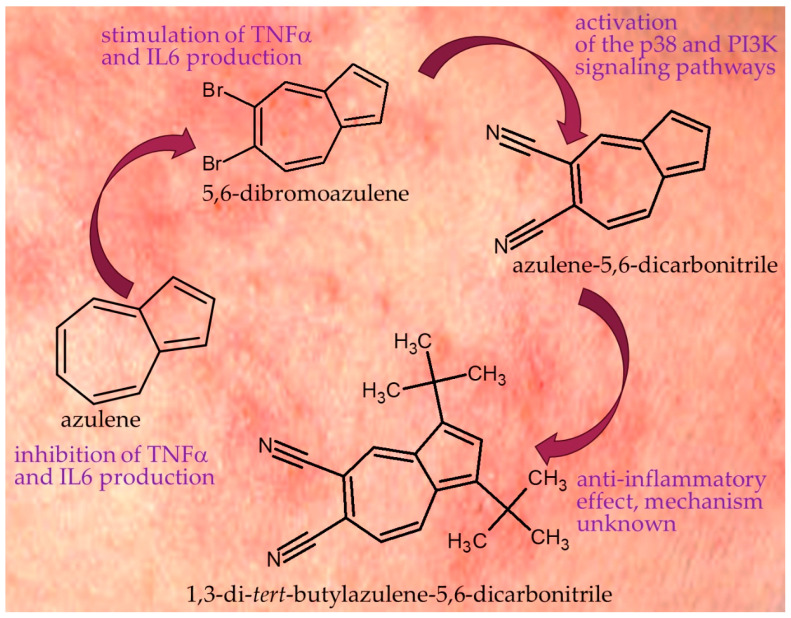
Azulene and its derivatives with anti-inflammatory effects on the skin.

In a study conducted in 2020, Furkan Ayaz et al. analyzed the impact of various azulene derivatives on lipopolysaccharide (LPS)-stimulated macrophages and assessed their immunostimulatory and anti-inflammatory activity. The study included azulene and two of its derivatives: 5,6-bromoazulene and 5,6-cyanoazulene (azulene-5,6-dicarbonitrile), as shown in Figure 2. In the case of 5,6-bromoazulene, it was found that this substance stimulates macrophages to produce pro-inflammatory cytokines such as tumor necrosis factor-α (TNF-α) and interleukin-6 (IL-6). Increasing the concentration of 5,6-bromoazulene to 10 μg/mL led to a significant production of TNF-α, without causing significant changes in cell viability. A dosage of 100 μg/mL resulted in decreased cell viability, leading to a lower level of cytokine production. 5,6-Bromoazulene also showed the ability to stimulate macrophages under conditions of no danger signal. In the case of 5,6-cyanoazulene, mild immunostimulatory activity was observed as well as a varied anti-inflammatory effect on LPS-stimulated macrophages, depending on the dosage. The highest concentration of 5,6-cyanoazulene caused a significant but slight decrease in cell viability while simultaneously stimulating TNF-α production. However, IL-6 production was more dependent on cell viability, and at higher concentrations, a decrease in the level of IL-6 was observed. In the case of unsubstituted azulene, no immunostimulatory activity was observed, but a strong anti-inflammatory effect was noted. Azulene inhibited the production of TNF-α and IL-6 by LPS-stimulated macrophages, without affecting their viability. Azulene derivatives exhibited comparable anti-inflammatory activity, but stronger than salicylic acid. 5,6-Bromoazulene may be considered as a potential candidate for an immunostimulatory drug, while 5,6-cyanoazulene may be used to suppress chronic inflammatory reactions. Unsubstituted azulene demonstrates strong anti-inflammatory activity without immunostimulatory effects [16,17].

In a scientific paper published in 2022 by Derya Yetkin et al., the properties of compounds including 5,6-cyanoazulene were also investigated. They presented interesting discoveries regarding the anti-inflammatory properties of two chemical compounds—the aforementioned 5,6-dicyanoazulene (azulene-5,6-dicarbonitrile) and 1,3-ditert-butylazulene-5,6-dicarbonitrile. Based on the conducted tests, it was demonstrated that 5,6-dicyanoazulene has the ability to counteract inflammatory processes through the activation of p38 and PI3K signaling pathways. Equally significant, the second compound examined, 1,3-ditert-butylazulene-5,6-dicarbonitrile, also exhibits anti-inflammatory properties, although the mechanism of its action remains unknown (Figure 2). An interesting aspect is that both azulene derivatives have been identified as potential candidates for photodynamic therapy drugs. This indicates their potential application in therapy, especially in the context of treating diseases associated with inflammatory processes [18,19].

In experiments conducted in 2020 by Ampika Phutim-Mangkhalthon et al., the efficacy of photodynamic therapy using guaiazulene and a red laser on peripheral blood cells was investigated to assess its anti-inflammatory effects. It was found that irradiated guaiazulene solutions generated more singlet oxygen and reduced the levels of inflammatory markers in peripheral blood cells. The results suggest that the photodynamic activation of guaiazulene may be an effective therapeutic method in treating inflammatory conditions such as oral mucosal inflammation [14].

**Figure 3:**
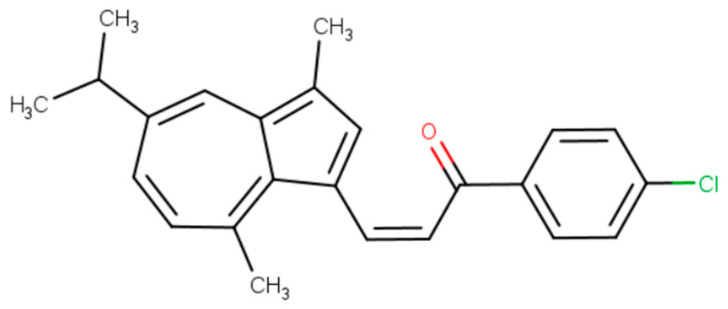
A new azulene derivative based on chalcone and guaiazulene: (2*Z*)-1-(4-chlorophenyl)-3-[3,8-dimethyl-5-(propan-2-yl)azulen-1-yl]prop-2-en-1-one with potential anti-inflammatory activity.

Zongchen Ma et al. synthesized derivatives of guaiazulene and examined them for anti-inflammatory activity. A series of chemical compounds—sulfonamides and chalcones based on guaiazulene—were utilized in the study. The experiment was conducted using a fluorescent macrophage transgenic model of *Danio rerio* (*D. rerio*), 3 days post-fertilization, with indomethacin as a positive control. Sulfonamide derivatives did not exhibit satisfactory anti-inflammatory activity, although sodium guaiazulene sulfonate is used in clinical practice [5,20]. On the other hand, guaiazulene-based chalcones exhibited significant anti-inflammatory activity, especially the compound presented in Figure 3, which showed a degree of inhibition of the inflammatory response in the zebrafish model at approximately 34%, surpassing the efficacy of indomethacin (approximately 27%) as a positive control. Additionally, a considerable reduction in the migration of inflammatory cells was observed after treatment with guaiazulene-based chalcones. These results suggest that the proposed compounds are promising candidates for further research as potential anti-inflammatory drugs [21].

## 4. Anti-UV Activity of Azulene Derivatives and Their Probable Mechanisms of Action

In experiments conducted in 2013 by Jun-Ichi et al., azulen-related compounds were analyzed for their photoprotective abilities against UV radiation. Sodium ascorbate served as the positive control in these experiments. The results indicated that one of the investigated compounds—benzo[b]cyclohepta[e][1,4]thiazine, in combination with sodium ascorbate—exhibited a synergistic protective effect. However, the insolubility of this compound in water posed a significant limitation for further research. Additionally, it was observed that a higher protective effect was observed in hematopoietic stem cells-3 (HSC-3) compared to human fibroblast cells [22].

**Figure 4:**
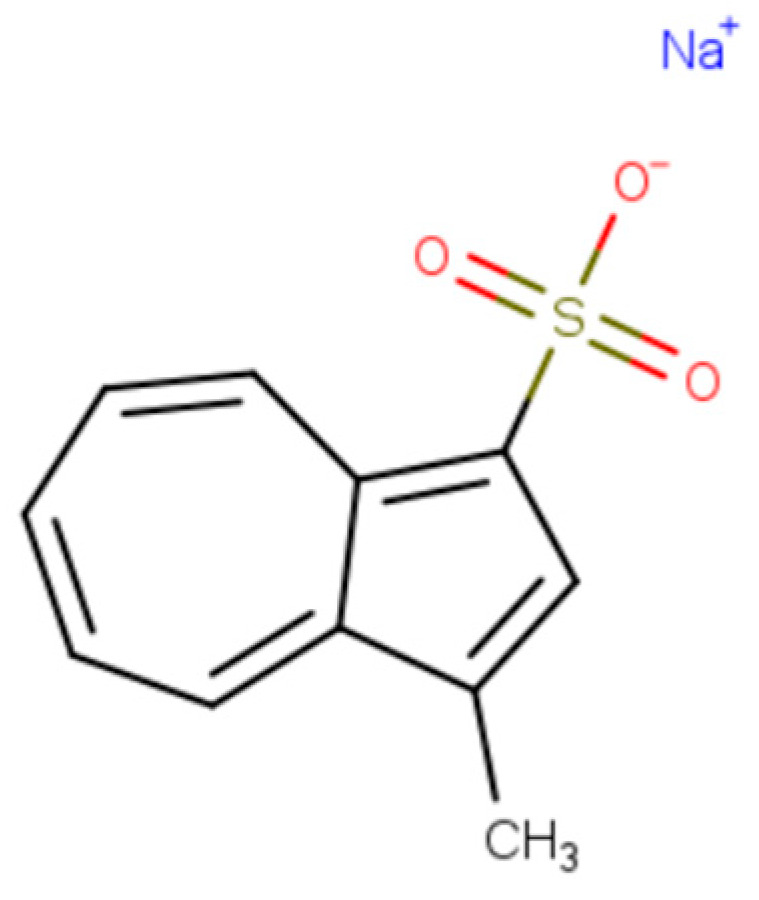
Sodium 3-methylazulene-1-sulfonate with anti-UV properties.

In 2013, the same researchers attempted to synthesize water-soluble derivatives of azulene. Among the nine synthesized salts, particular interest was drawn to the compound presented in Figure 4 due to its strongest ability to protect against UV radiation as well as its anticancer activity [23].

In the case of this compound, three potential mechanisms for inducing anti-UV activity are envisaged: the direct absorption of light at specific wavelengths, the scavenging of free radicals generated by UV radiation, and the inhibition of the lipooxygenase-1 (LOX-1) signaling pathway. Understanding these mechanisms is crucial for the further development of effective strategies for protection against harmful UV radiation. The results of the aforementioned studies indicate a high potential for the UV photoprotection of azulene derivatives and their potential anticancer activity. However, further research is necessary to verify the optimal conditions for the use of the proposed substances, taking into account their physicochemical properties and precise mechanisms of action, in order to develop preventive and therapeutic strategies [23,24,25].

In 2023, studies were conducted on the essential oil of *Artemisia sieversiana* Ehrh. ex Willd. (*A. sieversiana*) and its main component, chamazulene, regarding their photoprotective properties against the human keratinocyte cell line (HaCaT) and their effectiveness in protecting these cells from UVB radiation. The research not only demonstrated the anti-UVB activity of the analyzed raw material and chamazulene, but also their ability to alleviate oxidative stress and UVB-induced inflammatory response, suggesting their potential photoprotective and antioxidant properties in sun protection products, especially considering the synergy with chemical UVB filters [26,27,28,29].

## 5. Activity of Azulene Derivatives against Inflammatory Skin Diseases

**Scheme 1:**
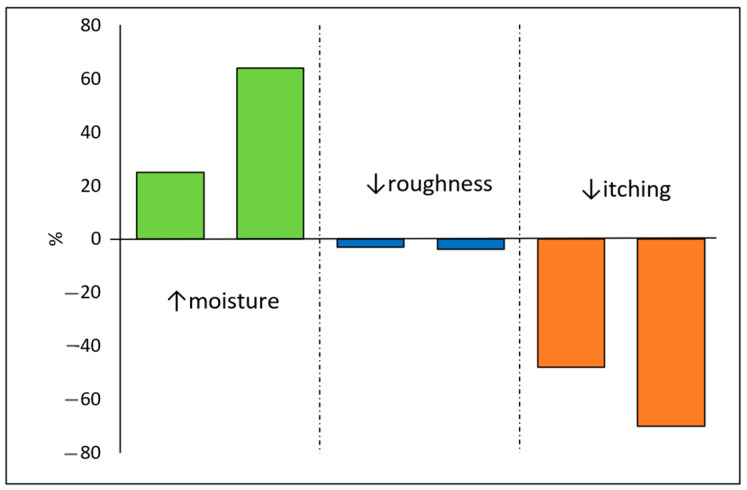
Assessment of skin moisture, roughness, and itching after 2 (A) and 4 (B) weeks of using guaiazulene with ceramides.

In light of current research on the use of azulene and its derivatives in the therapy of dermatological diseases, particularly focused on alleviating itching and atopic dermatitis, the presented clinical results offer a promising perspective for patients affected by these conditions [30]. Clinical studies conducted in 2022 involving 23 patients demonstrated that the use of 0.02% guaiazulene in combination with 1% ceramides significantly benefited the health and condition of the skin as well as markedly improved the sleep quality in patients with atopic dermatitis (AD). Regarding skin condition improvement, after 4 weeks of treatment, an impressive increase in skin moisture of approximately 64% was observed, along with a reduction in roughness by approximately 4%. In terms of skin health improvement, after 4 weeks of treatment, itching decreased by nearly 70%. A comparison of the monitored effects is presented in Scheme 1. Simultaneously with the improvement in skin parameters and its health in patients with AD, a significant enhancement in sleep quality of approximately 74% was observed [31].

The subsequent study evaluating the skin condition under the influence of 0.02% guaiazulene in combination with 2% ceramide NP showed a significant increase in skin moisture content by over 90% after 4 weeks of their application [32]. The analyzed tests demonstrate that the increase in ceramide concentration used in conjunction with guaiazulene contributes to the increase in skin moisture. Therefore, it would be beneficial to conduct comparative observations using a control group containing only ceramides without the involvement of azulene derivatives [33].

In 2021, other studies focused on evaluating the anti-inflammatory and antioxidant properties of guaiazulene compared to azulene and guaiazulene sulfonate. Using a micellar preparation, scientists conducted experiments including the enzyme-linked immunosorbent assay (ELISA), demonstrating that guaiazulene exhibited a broader spectrum of anti-inflammatory and antioxidant activity [34]. In another study, 0.05% guaiazulene was applied for resistant diaper dermatitis in newborns. The results confirmed a significant improvement as early as the first day of therapy, indicating the effectiveness of guaiazulene as an alternative form of treatment surpassing the standard methods. These findings provide promising evidence for the effectiveness of azulene and its derivatives as potential compounds in the therapy of dermatological diseases, opening new avenues for future research and therapeutic approaches in alleviating itching and atopic dermatitis [35,36,37].

## 6. Potential Anticancer Activity of Azulene Derivatives

**Figure 5:**
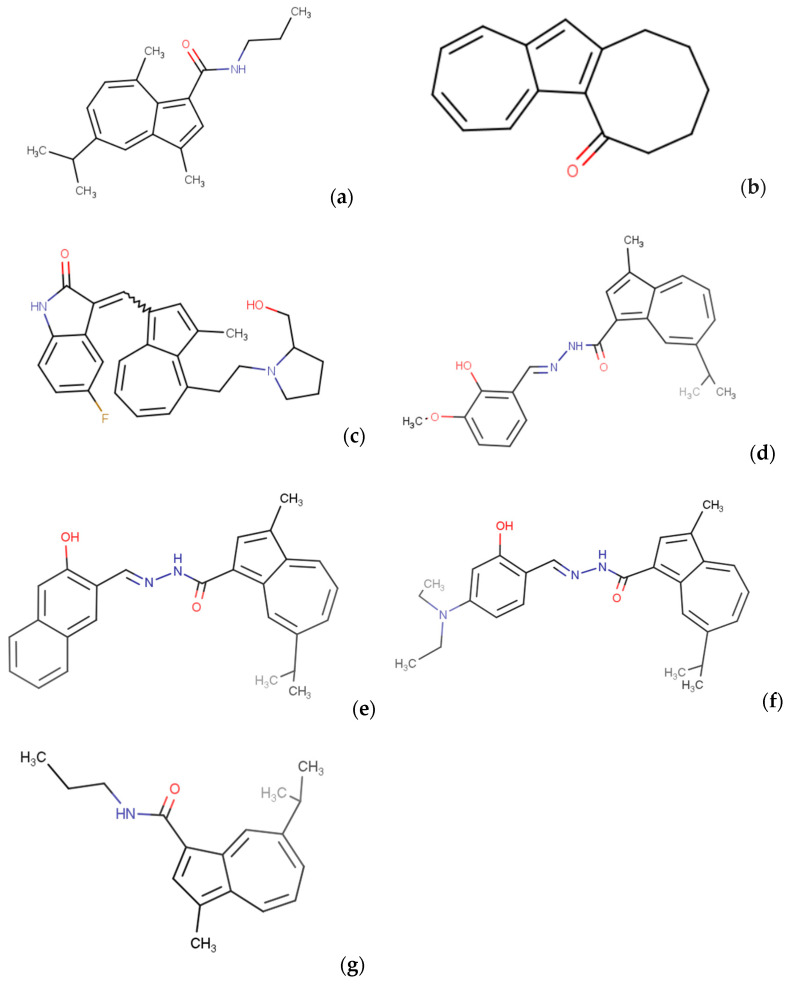
Structural formula of 3,8-dimethyl-5-(propan-2-yl)-*N*-propylazulene-1-carboxamide (**a**); 6*H*,7*H*,8*H*,9*H*,10*H*,11*H*-cycloocta[a]azulen-6-one (**b**); 5-fluoro-3-[(4-{2-[2-(hydroxymethyl)pyrrolidin-1-yl]ethyl}-3-methylazulen-1-yl)methylidene]-2,3-dihydro-1*H*-indol-2-one (**c**); *N*′-[(*E*)-(2-hydroxy-3-methoxyphenyl)methylidene]-3-methyl-7-(propan-2-yl)azulene-1-carbohydrazide (**d**); *N*′-[(*E*)-(3-hydroxynaphthalen-2-yl)methylidene]-3-methyl-7-(propan-2-yl)azulene-1-carbohydrazide (**e**); *N*′-[(*E*)-[4-(diethylamino)-2-hydroxyphenyl]methylidene]-3-methyl-7-(propan-2-yl)azulene-1-carbohydrazide (**f**); and 3-methyl-7-(propan-2-yl)-*N*-propylazulene-1-carboxamide (**g**), which are azulene derivatives with potential anticancer activity.

In studies published in 2018, the anticancer activity of 3,8-dimethyl-5-(propan-2-yl)-N-propylazulene-1-carboxamide derivative (Figure 5a) was determined. The cytotoxicity of this compound was evaluated against four human oral squamous cell carcinoma (OSCC) cell lines and three human normal oral cell lines. The analysis results indicated that the analyzed substance exhibited a higher tumor specificity (TS) value and partial selectivity strength (PSE) among the examined derivatives compared to doxorubicin. Quantitative structure–activity relationship (QSAR) analysis showed that the specificity of the Figure 5a derivative toward tumors was strongly correlated with hydrophobicity and molecular shape. In light of the obtained results, the analyzed derivative of guaiazulene demonstrates potential anticancer activity, which requires confirmation through further research. The ability of the analyzed substance to selectively act on cancer cells, induce apoptosis, and high TS and PSE values make it a promising direction in the search for effective anticancer drugs [38].

In another study by Chieko Kasami et al. in 2021, the focus was on seeking effective therapies targeting the energy metabolism of cancer cells. In the context of the resistance of cancer cells to molecularly targeted therapies, particular interest was drawn to 6H,7H,8H,9H,10H,11H-cycloocta[a]azulen-6-one, presented in Figure 5b. The mechanism of action of this substance was investigated both on cancer cell lines and in the *Caenorhabditis elegans* (*C. elegans*) animal model. The results suggest that the Figure 5b derivative acts on mitochondrial electron transport chain complex II, effectively inhibiting the process of oxidative phosphorylation, leading to the disruption of cellular energy production. The compound under investigation may be a promising candidate for a new anticancer agent capable of selectively targeting the mitochondria of cancer cells. Its ability to disrupt energy metabolism and induce apoptosis opens avenues for further research into its potential application in cancer therapy [39].

In experiments published in 2010, researchers synthesized a series of new azulene derivatives exhibiting inhibitory properties against tyrosine kinase receptors including fms-like tyrosine kinase 3 (FLT-3). Structural–activity relationship (SAR) analysis led to the modification of these potential substances, resulting in the identification of the most promising compound presented in Figure 5c. Studies revealed that this compound acts as an inhibitor of FLT-3. This discovery marks a significant advancement in the field of therapeutic tyrosine kinase inhibitors, particularly FLT-3, which play a crucial role in processes related to leukemia development. The new series of azulene derivatives present potential for further research into anticancer drugs, offering promising therapeutic prospects for more effective treatment of FLT-3-activated cancers. This finding underscores the significant role of azulene as a structural scaffold in designing drugs active against tyrosine kinase receptors [40].

The scientific publication from 2022 by Tereza Brogyányi et al. presents the results of research on three new derivatives of azulene hydrazide-hydrazones presented in Figure 5d–f. The compounds exhibited strong affinity toward iron(II) ions. Their anticancer activity was investigated on three pancreatic cancer cell lines as well as on healthy human fibroblasts BJh-TERT, demonstrating cytotoxic effects against pancreatic cancer cell lines. The compounds showed better selectivity toward cancer cells than healthy cells. The observed effects were linked to higher expression of NDRG1, hypoxia-inducible factor 1 (HIF-1α), and transferrin receptor protein 1 (TfR1) as well as the accumulation of the tested compounds in mitochondria and lysosomes, suggesting a mechanism of action based on the induction of mitophagy. Azulene hydrazide-hydrazones derivatives may represent a promising direction in designing potent and selective anticancer agents that have yet to be fully explored [41].

In a study conducted by Imanari K. et al. in 2019, the cytotoxic activity of 21 azulene amides was compared, among which one derivative, as presented in Figure 5g, proved to be the most promising. Their impact on oral squamous cell carcinoma cells and normal cells was investigated. The aforementioned derivative exhibited the highest specificity toward cancer cells, as confirmed by both the TS and PSE values. Analysis of the apoptosis expression suggests that the mechanism of action of the compound (Figure 5g) may be associated with pathways other than apoptosis induction. QSAR analysis revealed that anticancer specificity is correlated with the shape and lipophilicity of molecules. Moreover, the study suggests that some compounds may stimulate the growth of normal cells, which requires further investigation. These results underscore the importance of further research into potential anticancer drugs based on azulenes, and the need to identify more effective and specific anticancer azulene-type compounds [42].

## 7. The Activity of Azulene Derivatives in Erectile Dysfunction

**Figure 6:**
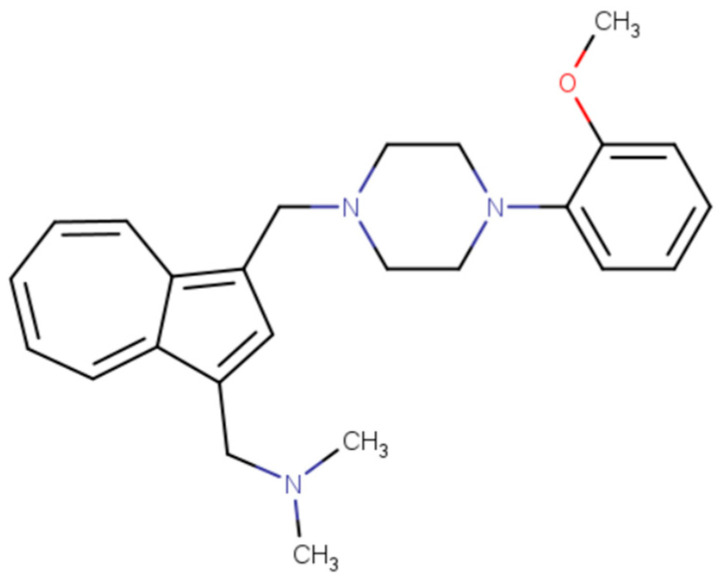
Structural formula of [(3-{[4-(2-methoxyphenyl)piperazin-1-yl]methyl}azulen-1-yl)methyl]dimethylamine, which is an azulene derivative with potential activity in erectile dysfunction.

In a study conducted by S. Löber et al. in 2012, azulene derivatives were investigated in the context of erectile dysfunction. As part of the experiment, a series of azulene derivatives were synthesized and their affinity to monoaminergic G protein-coupled receptors (GPCRs) was determined including dopamine, serotonin, histamine, and α-adrenergic receptors. Analysis of the activity of the most promising compounds revealed that the derivative presented in Figure 6 is a promising partial agonist of the D_4_ receptor (EC_50_ = 0.41 nM). The study assessed the compound’s ability to stimulate penile erection. Using an in vivo animal model in rats, the compound was found to effectively stimulate penile erection in males, exhibiting higher activity at low concentrations compared to apomorphine. The results obtained suggest that azulene derivatives, especially the one presented in Figure 6, may represent a promising substance for the development of drugs used in erectile dysfunction [43].

## 8. Limitations in the Use of Azulene and Its Derivatives on the Skin

The potential use of azulene and guaiazulene on the skin as cosmetic ingredients raises both hopes and concerns. Studies suggest that these compounds, known for their antioxidant properties, may neutralize reactive oxygen species (ROS) generated during UV radiation exposure. However, analysis of their mechanisms of action has shown that the photodecomposition of azulene and guaiazulene under the influence of UVA radiation can lead to the formation of various products including oligomeric polyoxygenated compounds [44,45]. Importantly, lipid peroxidation induction and the generation of ROS such as carbon-centered radicals and peroxide radicals pose potential threats to skin health. There is a risk of DNA damage, DNA adduct formation, and even potential impact on tumor development in experimental animals [46,47]. In the context of prolonged use, especially with concurrent UV light exposure, further research is needed to gain a better understanding of the biological effects of azulene and guaiazulene derivatives. Retinyl palmitate, a well-known and widely used compound in dermatology, esthetic medicine, and cosmetology, which is a more stable form of retinol, also generates ROS upon exposure to UVA light including superoxide anion radicals, singlet oxygen, and other reactive chemical species. These, in turn, are known for their ability to induce lipid peroxidation, potentially leading to cell damage including skin cells [48,49,50,51]. Therefore, when using azulene and guaiazulene derivatives for their dermato-cosmetic and dermato-therapeutic benefits, caution should be exercised, taking into account the potential risks associated with photodecomposition and ROS generation [44,46,47,52]. It is worth noting that in the absence of UV light, azulene and guaiazulene do not exhibit mutagenicity [5]. The perspective for future research may involve the search for azulene derivatives characterized by greater photostability and the absence of phototoxicity. Structural optimization of these compounds should lead to derivatives that retain their beneficial skincare and therapeutic properties while reducing the risk of ROS generation under UV light exposure. Studies on new derivatives should focus on increasing photostability and minimizing side effects to ensure the safe use of these substances in dermo-therapeutic and dermo-cosmetic applications [53,54,55]. Introducing photostable derivatives of azulene to the market could open up new perspectives for the cosmetic, pharmaceutical, and medical industries [5].

## 9. Azulene-Based Polymers

**Figure 7:**
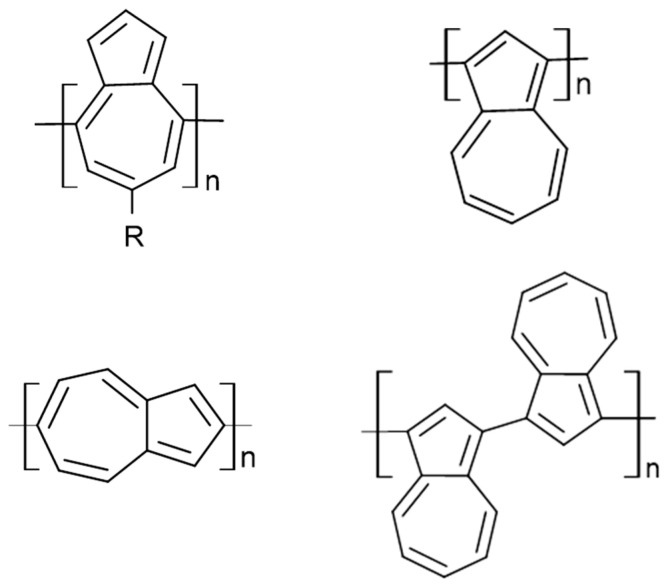
Examples of monomers containing azulene in their structure.

Azulene-based polymeric compounds represent a promising class of materials for pharmaceutical applications. Their unique chemical structure and physical properties such as color, photo-stimulability, and responsiveness to light stimuli have attracted the attention of researchers in the quest for new therapeutic solutions. Methods for synthesizing these polymers include both chemical and electrochemical reactions, enabling the controlled construction of macromolecules and the introduction of desired functionalities. Monomers can be arranged in cyclic or linear structures, with or without substituents, and examples of monomers are shown in Figure 7. In pharmaceuticals, azulene polymers hold potential as drug carriers, allowing for the modulation of their physicochemical properties to optimally deliver active substances. Additionally, their ability to generate reactive oxygen species upon light exposure makes them suitable for photodynamic therapy, particularly in cancer treatment. Furthermore, due to their potential anti-inflammatory and antibacterial effects, azulene polymers may find application in the treatment of skin conditions such as acne or psoriasis. Finally, their potential for drug transport across biological barriers suggests their utility in the therapy of neurological diseases or in delivering active substances to specific target sites in the body [9,10,11,12,56].

## 10. Perspective for Further Research

**Figure 8:**
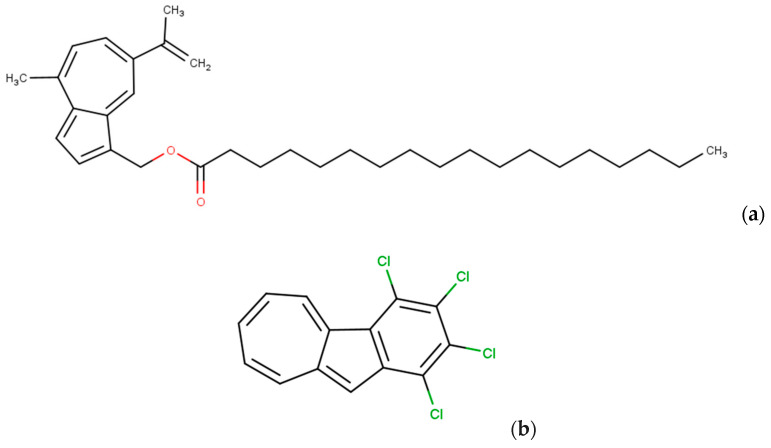
Structural formula of [4-methyl-7-(prop-1-en-2-yl)azulen-1-yl]methyl octadecenoate (**a**) and 1,2,3,4-tetrachlorobenzo[a]azulene (**b**) presenting exemplary chemical compounds as a perspective for further research in terms of therapy.

In 2023, Maruoka K. et al. isolated a derivative of azulene from the fungus *L. indigo* and subjected it to modifications as depicted in Figure 8a. It was synthesized with the aim to find a blue food dye in an aqueous dispersion using Tween 80. A drawback of this solution may be the bitter taste of the solubilizer, and additionally, azulene itself has an intense odor, which may be less intense in the case of its derivatives. These aspects may affect the odor or taste of the food. A bitter taste is not a problem in the context of therapy or skincare, hence such derivatives could be investigated for their potential properties in medical applications [57].

In the scientific article by García-Martínez et al. in 2024, a series of new phenethylamine-azulene conjugates was synthesized. Researchers focused on the mechanism regulating the opening of the aziridine ring. The β-aryloethylamine scaffold plays a significant role in medicinal chemistry due to the abundance of naturally occurring and synthetic derivatives exhibiting diverse pharmacological activities. This may represent another perspective for further investigation regarding skincare or therapy [58].

The researcher A.C. Razus also discussed techniques for synthesizing benz[a]azulenes, condensed azulenes as well as helicenes or nanographenes with azulene and other substrates, thus opening up new possibilities for the synthesis and investigation of potential anticancer agents and therapy for skin diseases. An example of the new derivative is shown in Figure 8b [59].

## 11. Summary

Azulene and its derivatives demonstrate promising anti-inflammatory properties, which create new therapeutic perspectives in dermatology. Structural modifications of azulene can influence various biological effects such as stimulating the production of pro-inflammatory cytokines or immunostimulatory activity. The wide range of applications for azulene derivatives includes UV protection, alleviating atopic dermatitis and itching as well as treating erectile dysfunction and potential anticancer activity. However, there are limitations in the use of unformulated azulene on the skin, especially related to photodecomposition and the generation of reactive oxygen species under UV radiation, which requires further research into the safety of these substances in long-term use or their application in phototherapy. In summary, this article provides an overview of the current achievements regarding the activity of azulene and its derivatives, outlining the therapeutic and skincare potential of this group of substances in dermatology, while emphasizing the need for further research into their safety and efficacy.

## Data Availability

Not applicable.

## References

[1] Zheng J.-J., Shao C.-L., Chen M., Gan L.-S., Fang Y.-C., Wang X.-H., Wang C.-Y. (2014). Ochracenoids A and B, Guaiazulene-Based Analogues from Gorgonian Anthogorgia Ochracea Collected from the South China Sea. Mar. Drugs.

[2] Chen D., Yu S., van Ofwegen L., Proksch P., Lin W. (2012). Anthogorgienes A–O, New Guaiazulene-Derived Terpenoids from a Chinese Gorgonian Anthogorgia Species, and Their Antifouling and Antibiotic Activities. J. Agric. Food Chem..

[3] Podkowa A., Kryczyk-Poprawa A., Opoka W., Muszyńska B. (2021). Culinary–Medicinal Mushrooms: A Review of Organic Compounds and Bioelements with Antioxidant Activity. Eur. Food Res. Technol..

[4] Nicholas G.M. (1998). Australasian Fungi: A Natural Product Study. Ph.D. Thesis.

[5] Akram W., Tagde P., Ahmed S., Arora S., Emran T.B., Babalghith A.O., Sweilam S.H., Simal-Gandara J. (2023). Guaiazulene and Related Compounds: A Review of Current Perspective on Biomedical Applications. Life Sci..

[6] Höferl M., Wanner J., Tabanca N., Ali A., Gochev V., Schmidt E., Kaul V.K., Singh V., Jirovetz L. (2020). Biological Activity of Matricaria Chamomilla Essential Oils of Various Chemotypes. Planta Medica Int. Open.

[7] Veys K., Escudero D. (2020). Computational Protocol To Predict Anti-Kasha Emissions: The Case of Azulene Derivatives. J. Phys. Chem. A.

[8] Patiño Cano L.P., Quintana Manfredi R., Pérez M., García M., Blustein G., Cordeiro R., Pérez C.D., Schejter L., Palermo J.A. (2018). Isolation and Antifouling Activity of Azulene Derivatives from the Antarctic Gorgonian Acanthogorgia Laxa. Chem. Biodivers..

[9] Zeng H.N., Png Z.M., Xu J. (2020). Azulene in Polymers and Their Properties. Chem.–Asian J..

[10] Shetti V.S. (2021). Chemical Syntheses and Salient Features of Azulene-Containing Homo- and Copolymers. Beilstein J. Org. Chem..

[11] Razus A.C. (2022). Dancing with Azulene. Symmetry.

[12] Liu R., Fu Y., Wu F., Liu F., Zhang J.-J., Yang L., Popov A.A., Ma J., Feng X. (2023). Modular Synthesis of Structurally Diverse Azulene-Embedded Polycyclic Aromatic Hydrocarbons by Knoevenagel-Type Condensation. Angew. Chem. Int. Ed..

[13] Ou L., Zhou Y., Wu B., Zhu L. (2019). The Unusual Physicochemical Properties of Azulene and Azulene-Based Compounds. Chin. Chem. Lett..

[14] Phutim-Mangkhalthon A., Teerakapong A., Tippayawat P., Morales N.P., Morkmued S., Puasiri S., Priprem A., Damrongrungruang T. (2020). Anti-Inflammatory Effect of Photodynamic Therapy Using Guaiazulene and Red Lasers on Peripheral Blood Mononuclear Cells. Photodiagnosis Photodyn. Ther..

[15] Srivastava J.K., Pandey M., Gupta S. (2009). Chamomile, a Novel and Selective COX-2 Inhibitor with Anti-Inflammatory Activity. Life Sci..

[16] Ayaz F., Yuzer A., Ince T., Ince M. (2020). Anti-Cancer and Anti-Inflammatory Activities of Bromo- and Cyano-Substituted Azulene Derivatives. Inflammation.

[17] Xiao M., Gao X. (2023). Design, Synthesis and Anti-Inflammatory Activity of Azulene Derivatives Containing Benzimidazole Unit. Chin. J. Org. Chem..

[18] Yetkin D., Ince T., Ayaz F. (2022). Photodynamic Anti-Inflammatory Activity of Azulene Derivatives on Mammalian Macrophages and Their Intracellular Mechanism of Action. Photodiagnosis Photodyn. Ther..

[19] Alim F.G., Hayuningtyas R.A. (2023). Potensi Chamomile Sebagai Agen Antiinflamasi Oral. J. Kedokt. Gigi Terpadu.

[20] Uesugi A., Tsushima F., Miyamoto Y., Harada H. (2023). Pollen Food Allergy Syndrome Caused by Japanese Radish: A Case Report. Indian J. Dermatol..

[21] Ma Z., Han X., Ren J., Liu K., Zhang W., Li G. (2023). Design, Synthesis, and Biological Activity of Guaiazulene Derivatives. Chem. Biodivers..

[22] Ueki J.-I., Shimada A., Sakagami H., Wakabayashi H. (2011). Hormetic and UV-Protective Effects of Azulene-Related Compounds. In Vivo.

[23] Ueki J.-I., Sakagami H., Wakabayashi H. (2013). Anti-UV Activity of Newly-Synthesized Water-Soluble Azulenes. In Vivo.

[24] Sakagami H., Sheng H., Okudaira N., Yasui T., Wakabayashi H., Jia J., Natori T., Suguro-Kitajima M., Oizumi H., Oizumi T. (2016). Prominent Anti-UV Activity and Possible Cosmetic Potential of Lignin-Carbohydrate Complex. In Vivo.

[25] Rekka E., Chrysselis M., Siskou I., Kourounakis A. (2002). Synthesis of New Azulene Derivatives and Study of Their Effect on Lipid Peroxidation and Lipoxygenase Activity. Chem. Pharm. Bull. (Tokyo).

[26] Zhou Y., He L., Wang W., Wei G., Ma L., Liu H., Yao L. (2023). Artemisia Sieversiana Ehrhart Ex Willd. Essential Oil and Its Main Component, Chamazulene: Their Photoprotective Effect against UVB-Induced Cellular Damage and Potential as Novel Natural Sunscreen Additives. ACS Sustain. Chem. Eng..

[27] Ma L., Xu J., Wang W., Lu J., Li Y., Yao L. (2023). Thin Layer Chromatography-Direct Bioautography for Investigation of Antibacterial Activities of Artemisia Sieversiana Ehrhart Ex Willd. Essential Oil. Nat. Prod. Res..

[28] Xiao T., Chen Y., Song C., Xu S., Lin S., Li M., Chen X., Gu H. (2021). Possible Treatment for UVB-Induced Skin Injury: Anti-Inflammatory and Cytoprotective Role of Metformin in UVB-Irradiated Keratinocytes. J. Dermatol. Sci..

[29] Ghasemi M., Babaeian Jelodar N., Modarresi M., Bagheri N., Jamali A. (2016). Increase of Chamazulene and α-Bisabolol Contents of the Essential Oil of German Chamomile (*Matricaria chamomilla* L.) Using Salicylic Acid Treatments under Normal and Heat Stress Conditions. Foods.

[30] Kobayashi Y., Nakano Y., Inayama K., Sakai A., Kamiya T. (2003). Dietary Intake of the Flower Extracts of German Chamomile (*Matricaria recutita* L.) Inhibited Compound 48/80-Induced Itch-Scratch Responses in Mice. Phytomedicine.

[31] Park S.I., Lee K.W., Park S., Shin M.S. (2022). Itching and Atopy Relief Using Azulene Derivatives and High-Content Ceramide Skin Barrier Nano-Liposome Structures by Dry Skin. Int. J. Intell. Syst. Appl. Eng..

[32] Park S.I., Lee J., Shin M.S., Pattnaik P.K., Vaidya A., Mohanty S., Mohanty S., Hol A. (2022). Clinical Study on Itching Relief Caused by Dry Skin of Cosmetics Containing Ceramide NP and Guaiazulene in Smart Healthcare Products. Smart Healthcare Analytics: State of the Art.

[33] Mahato R.K., Singh M., Pathak H., Gogoi N.R., Kharbithai R., Chowrasia P., Bora P.L., Sarkar T., Jana B.K., Mazumder B. (2023). Emerging Nanotechnology Backed Formulations for the Management of Atopic Dermatitis. Ther. Deliv..

[34] Park S.I., Lee K.W., Park S., Shin M.S. (2021). In Vitro Biological Activities of Azulene, Guaiazulene, and Sodium Guaiazulene Sulfonate and Its Application to Formulations through PEG-PCL Micelles. RIGEO.

[35] Bakun P., Czarczynska-Goslinska B., Goslinski T., Lijewski S. (2021). In Vitro and in Vivo Biological Activities of Azulene Derivatives with Potential Applications in Medicine. Med. Chem. Res..

[36] Gunes T., Akin M.A., Sarici D., Hallac K., Kurtoglu S., Hashimoto T. (2013). Guaiazulene: A New Treatment Option for Recalcitrant Diaper Dermatitis in NICU Patients. J. Matern.-Fetal Neonatal Med. Off. J. Eur. Assoc. Perinat. Med. Fed. Asia Ocean. Perinat. Soc. Int. Soc. Perinat. Obstet..

[37] Furau C., Grossmann H., Furau G., Vormann J. (2016). Effect of a Guaiazulene-Containing Ointment on Nipple and Areola Area Health of Women. J. Cosmet. Dermatol. Sci. Appl..

[38] Wada T., Maruyama R., Irie Y., Hashimoto M., Wakabayashi H., Okudaira N., Uesawa Y., Kagaya H., Sakagami H. (2018). In Vitro Anti-Tumor Activity of Azulene Amide Derivatives. In Vivo Athens Greece.

[39] Kasami C., Yamaguchi J.-I., Inoue H. (2021). Guaiazulene Derivative 1,2,3,4-Tetrahydroazuleno[1,2-b] Tropone Reduces the Production of ATP by Inhibiting Electron Transfer Complex II. FEBS Open Bio.

[40] Chen C.-H., Lee O., Yao C.-N., Chuang M.-Y., Chang Y.-L., Chang M.-H., Wen Y.-F., Yang W.-H., Ko C.-H., Chou N.-T. (2010). Novel Azulene-Based Derivatives as Potent Multi-Receptor Tyrosine Kinase Inhibitors. Bioorg. Med. Chem. Lett..

[41] Brogyányi T., Kaplánek R., Kejík Z., Hosnedlová B., Antonyová V., Abramenko N., Veselá K., Martásek P., Vokurka M., Richardson D.R. (2022). Azulene Hydrazide-Hydrazones for Selective Targeting of Pancreatic Cancer Cells. Biomed. Pharmacother..

[42] Imanari K., Hashimoto M., Wakabayashi H., Okudaira N., Bandow K., Nagai J., Tomomura M., Tomomura A., Uesawa Y., Sakagami H. (2019). Quantitative Structure–Cytotoxicity Relationship of Azulene Amide Derivatives. Anticancer. Res..

[43] Löber S., Hübner H., Buschauer A., Sanna F., Argiolas A., Melis M.R., Gmeiner P. (2012). Novel Azulene Derivatives for the Treatment of Erectile Dysfunction. Bioorg. Med. Chem. Lett..

[44] Chiang H.-M., Yin J.-J., Xia Q., Zhao Y., Fu P.P., Wen K.-C., Yu H. (2010). Photoirradiation of Azulene and Guaiazulene—Formation of Reactive Oxygen Species and Induction of Lipid Peroxidation. J. Photochem. Photobiol. Chem..

[45] Struwe M., Csato M., Singer T., Gocke E. (2011). Comprehensive Assessment of the Photomutagenicity, Photogenotoxicity and Photo(Cyto)Toxicity of Azulene. Mutat. Res. Toxicol. Environ. Mutagen..

[46] Wang L., Yan J., Fu P.P., Parekh K.A., Yu H. (2003). Photomutagenicity of Cosmetic Ingredient Chemicals Azulene and Guaiazulene. Mutat. Res..

[47] Wang L., Yan J., Wang S., Cohly H., Fu P.P., Hwang H.-M., Yu H. (2004). Phototoxicity and DNA Damage Induced by the Cosmetic Ingredient Chemical Azulene in Human Jurkat T-Cells. Mutat. Res..

[48] Xia Q., Yin J.J., Cherng S.-H., Wamer W.G., Boudreau M., Howard P.C., Fu P.P. (2006). UVA Photoirradiation of Retinyl Palmitate--Formation of Singlet Oxygen and Superoxide, and Their Role in Induction of Lipid Peroxidation. Toxicol. Lett..

[49] Mei N., Xia Q., Chen L., Moore M.M., Fu P.P., Chen T. (2005). Photomutagenicity of Retinyl Palmitate by Ultraviolet A Irradiation in Mouse Lymphoma Cells. Toxicol. Sci..

[50] Ibrahim T., Rouby M.N.E., Al-Sherbini E.-S.A.M., Noury A.H.E., Morsy M.E. (2016). Photodecomposition, Photomutagenicity and Photocytotoxicity of Retinyl Palmitate under He–Ne Laser Photoirradiation and Its Effects on Photodynamic Therapy of Cancer Cells in Vitro. Photodiagnosis Photodyn. Ther..

[51] Yan J., Xia Q., Cherng S.-H., Wamer W.G., Howard P.C., Yu H., Fu P.P. (2005). Photo-Induced DNA Damage and Photocytotoxicity of Retinyl Palmitate and Its Photodecomposition Products. Toxicol. Ind. Health.

[52] Damrongrungruang T., Kitchindaopat N., Thanasothon P., Theeranut K., Tippayawat P., Ruangsuwan C., Suwannee B. (2018). Effects of Photodynamic Therapy with Azulene on Peripheral Blood Mononuclear Cell Viability and Singlet Oxygen Formation. Photodiagnosis Photodyn. Ther..

[53] Leino T.O., Sieger P., Yli-Kauhaluoma J., Wallén E.A.A., Kley J.T. (2022). The Azulene Scaffold from a Medicinal Chemist’s Perspective: Physicochemical and in Vitro Parameters Relevant for Drug Discovery. Eur. J. Med. Chem..

[54] Kurihara T., Noguchi M., Noguchi T., Wakabayashi H., Motohashi N., Sakagami H. (2006). Relationship between Electronic Structure and Cytotoxic Activity of Azulenes. In Vivo.

[55] Damrongrungruang T., Rattanayatikul S., Sontikan N., Wuttirak B., Teerakapong A., Kaewrawang A. (2021). Effect of Different Irradiation Modes of Azulene-Mediated Photodynamic Therapy on Singlet Oxygen and PGE2 Formation. Photochem. Photobiol..

[56] Tsuchiya T., Higashibeppu M., Mazaki Y. (2023). Synthesis and Properties of Twisted and Helical Azulene Oligomers and Azulene-Based Polycyclic Hydrocarbons. ChemistryOpen.

[57] Maruoka K., Suzuki R., Kamishima T., Koseki Y., Ngoc Dao A.T., Murafuji T., Kasai H. (2023). Total Synthesis of Azulene Derivative, a Blue Pigment Isolated from Lactarius Indigo, and Colorant Application of Its Aqueous Dispersion. J. Agric. Food Chem..

[58] García-Martínez P., Bernardo O., Borge J., González J., López L.A. (2024). Synthesis of Phenethylamine-Azulene Conjugates Enabled by Regioselective Ring Opening of Aziridines. Adv. Synth. Catal..

[59] Razus A.C. (2024). Syntheses of Azulene Embedded Polycyclic Compounds. Symmetry.

